# Should Hebbian learning be selective for negative excess kurtosis?

**DOI:** 10.1186/1471-2202-16-S1-P65

**Published:** 2015-12-18

**Authors:** Claudius Gros, Samuel Eckmann, Rodrigo Echeveste

**Affiliations:** 1 Institute for Theoretical Physics, Goethe University, Frankfurt, 111932 , Germany

## 

Within the Hebbian learning paradigm, synaptic plasticity results in potentiation whenever pre- and postsynaptic activities are correlated, and in depression otherwise. This requirement is however not sufficient to determine the precise functional form for Hebbian learning, and a range of distinct formulations have been proposed hitherto. They differ, in particular, in the way runaway synaptic growth is avoided; by either imposing a hard upper bound for the synaptic strength, overall synaptic scaling, or additive synaptic decay [1]. Here we propose [2] a multiplicative Hebbian learning rule which is, at the same time, self-limiting and selective for negative excess kurtosis (for the case of symmetric input distributions).

**Figure 1:**
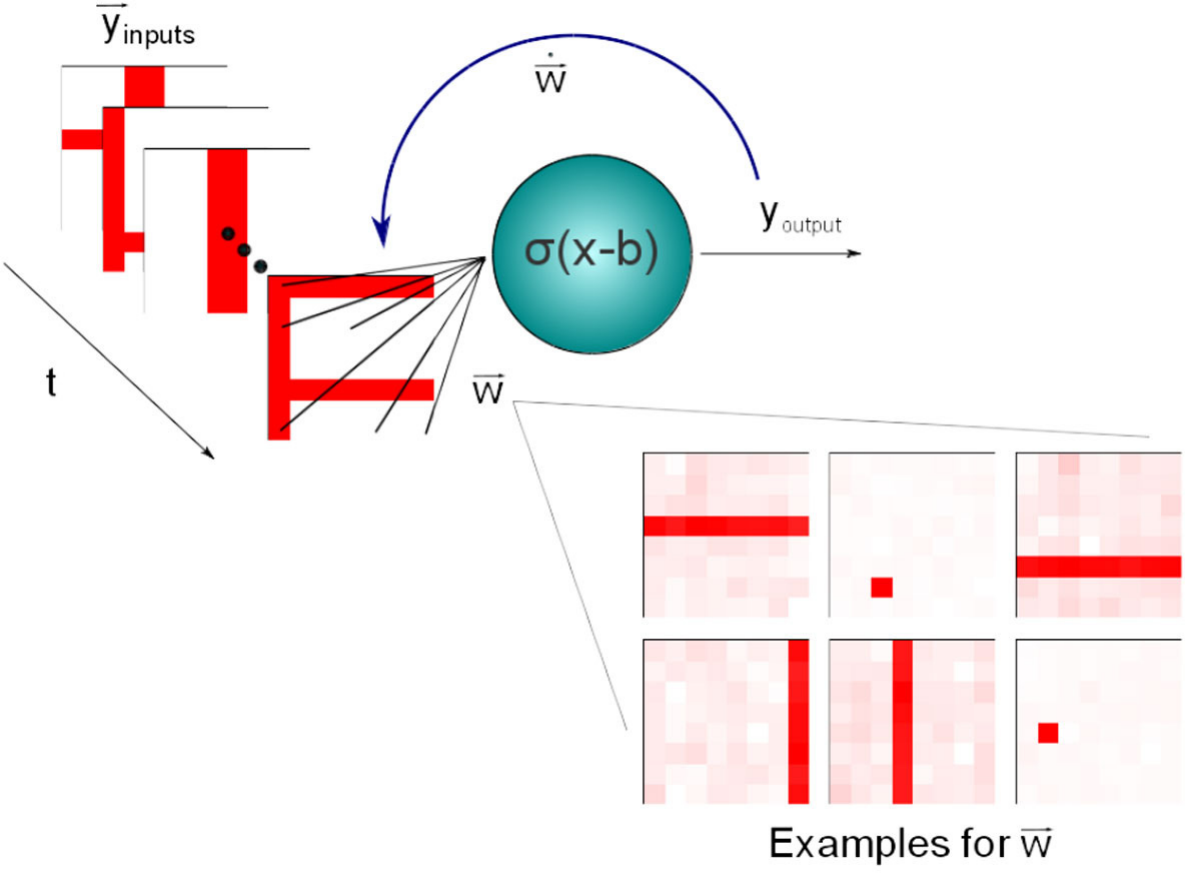
**The learning rule's ability to perform an independent component analysis is tested with the non-linear bars problem**. An input set consisting of a random number of horizontal and vertical bars is fed to the neuron which, after training, becomes selective to individual bars or pixels, the independent components of the input set, even when such an input was never presented to the neuron in isolation.

Hebbian learning results naturally in a principal component analysis (PCA), whenever one is present. Alternative formulations of the Hebbian learning paradigm differ however in other properties. Importantly they may, or may not, perform an independent component analysis (ICA), whenever one is feasible. The ICA may be achieved by maximizing (minimizing) the excess kurtosis, whenever the latter is positive (negative) [3]. Noting that naturally occurring individual cell lifetime firing rates are, however, characterized by having both a large kurtosis and a large skewness [4], we investigate in this paper the effect of the skewness and kurtosis on performing both PCA and ICA (see Figure 1) with several Hebbian learning rules. A particular emphasis is placed on the differences between additive and multiplicative schemes. We find that multiplicative Hebbian plasticity rules select both for small excess kurtosis and large skewness, allowing them to perform an ICA, in contrast to additive rules.
